# Lymph node ratio determines the benefit of adjuvant radiotherapy in pathologically 3 or less lymph node-positive prostate cancer after radical prostatectomy: a population-based analysis with propensity-score matching

**DOI:** 10.18632/oncotarget.22610

**Published:** 2017-11-22

**Authors:** Yi-Jun Kim, Changhoon Song, Keun-Yong Eom, In Ah Kim, Jae-Sung Kim

**Affiliations:** ^1^ Department of Radiation Oncology, Seoul National University College of Medicine, Seoul National University Bundang Hospital, Seongnam, Republic of Korea

**Keywords:** adjuvant radiotherapy, lymph node ratio, positive lymph node, prostate cancer, radical prostatectomy

## Abstract

**Background:**

The survival benefit of adjuvant radiotherapy (ART) in prostate adenocarcinoma, with limited numbers of pathologically involved lymph nodes (LNs) after radical prostatectomy (RP), is controversial.

**Materials and Methods:**

From 2004 to 2014, data for prostate cancer patients categorized as N1M0 after RP were retrieved from the Surveillance, Epidemiology, and End Results (SEER) database. After propensity-score matching, the 10-year cancer-specific survival (CSS) rates between patients who received ART (ART group) or did not/unknown (no-ART group) were compared for each stratum of lymph node ratio (LNR) (%) according to the number of involved LNs.

**Results:**

Optimal matching formed pairs of no-ART (*n* = 905) and ART (*n* = 905) groups. ART increased the CSS rate, even in patients with up to 3 positive LNs when the LNR is 7% or higher.

**Conclusions:**

ART after RP showed a CSS benefit in prostate adenocarcinoma with 4 or more involved LNs irrespective of LNR. In prostate adenocarcinoma with up to 3 involved LNs after RP, ART may provide CSS benefits when the LNR is 7% or higher. The number of LN dissections required to achieve an LNR below 7% is 15, 29, and 43 or more for 1, 2, and 3 involved LNs, respectively.

## INTRODUCTION

In 2017, prostate cancer accounted for 1 in 5 new cancer diagnoses and was the third leading cause of cancer death in men within the United States [1]. Even though most patients who undergo radical prostatectomy (RP) are cured of the disease, the 10-year progression-free survival rate decreases by 20–64% in patients with lymph node (LN) metastases [2–4]. Prostate-specific antigen (PSA) and Gleason scores have been identified as independent prognostic factors in prostate cancer, and are determining factors in current treatment strategies [5, 6]. In this PSA era, however, TNM stages are still useful prognostic indicators, and the N category has been found to be one of the strongest prognostic factors for non-distant metastatic prostate cancer patients [7, 8].

Although some studies indicate that adjuvant radiotherapy (ART) after RP increases survival in patients with LN involvements [9, 10], the role of ART in cases of a limited number of LN involvements remains controversial. Several studies have demonstrated that the prognosis in prostate cancer patients can be stratified according to the number of metastatic LNs [2, 11–15]. Specifically, for patients with a limited number of LN involvements, the survival benefit of ART was reduced or confined to patients with other high-risk factors such as high T categories or Gleason scores [15, 16]. In contrast, it has been suggested that ART has a survival benefit regardless of the number of metastatic LNs [10, 17].

The number of dissected LNs is also correlated with the treatment outcomes. Extended pelvic LN dissection appears to improve the cancer-specific survival (CSS) and progression-free survival rates in patients with LN involvements [14, 18, 19]. Therefore, it is plausible that the survival benefit from ART might depend on the LN ratio (LNR, defined as 100% × [number of involved LNs]/[number of dissected LNs]) [13]. Until now, the survival benefit from ART, stratified based on the number of involved LNs and the LNR, has not been fully evaluated.

With the development of effective systemic treatments, including androgen-depravation therapy (ADT), and the identification of biomarkers, such as PSA, as sensitive follow-up tools, some physicians are reluctant to administer ART. Instead, they prefer prompt salvage radiotherapy (RT) after biochemical failure to prevent overtreatment [20, 21]. Therefore, the development of detailed criteria for ART in patients with involved LNs after RP is crucial.

The Surveillance, Epidemiology, and End Results (SEER) database has documented PSA and Gleason score information since 2004. In this study, we examined a population-based cohort (using data from 2004 to 2014) of patients with prostate adenocarcinoma to investigate the benefits of ART. Subgroup analyses according to the number of involved LNs and the LNRs were performed to identify patients who were most likely to benefit from ART.

## RESULTS

### Patient characteristics before propensity-score matching

A total of 3548 patients satisfied the inclusion and exclusion criteria. The number of patients in the ART and no-ART groups was 905 and 2643, respectively. The comparison of patient characteristics between the ART and no-ART groups is summarized in Table 1. The ART group had a high proportion of patients with T3–4 categories (*p* < 0.000001), PSA ≥ 20 ng/mL (*p* = 0.011), and a Gleason score ≥ 8 (*p* = 0.000146) compared with the no-ART group. Meanwhile, the patients aged 65 years and older (*p* < 0.000001), and those with more than 10 LN dissections (*p* = 0.010) were less likely to receive ART.

**Table 1:** Characteristics of prostate adenocarcinoma patients after radical prostatectomy before and after propensity-score matching

Characteristics	Before propensity-score matching					After propensity-score matching				
	No-ART(*n* = 2643)		ART(*n* = 905)		*P* value^*^	No-ART(*n* = 905)		ART(*n* = 905)		*P* value^*^
	no.	(%)	no.	(%)		no.	(%)	no.	(%)	
**Age**										
< 65	1538	(58.2)	616	(68.1)	< 0.000001	617	(68.2)	616	(68.1)	0.960
≥ 65	1105	(41.8)	289	(31.9)		288	(31.8)	289	(31.9)	
Race										
White	2166	(82.0)	758	(83.8)	0.075	731	(80.8)	758	(83.8)	0.010
Black	369	(14.0)	102	(11.3)		142	(15.7)	102	(11.3)	
Others	108	(4.1)	45	(5.0)		32	(3.5)	45	(5.0)	
Year of diagnosis										
2004–2009	1121	(42.4)	377	(41.7)	0.691	344	(38.0)	377	(41.7)	0.113
2010–2014	1522	(57.6)	528	(58.3)		561	(62.0)	528	(58.3)	
T category										
1–2	559	(21.2)	112	(12.4)	< 0.000001	128	(14.1)	112	(12.4)	0.267
3–4	2084	(78.8)	793	(87.6)		777	(85.9)	793	(87.6)	
Grade										
1–2	228	(8.6)	63	(7.0)	0.115	65	(7.2)	63	(7.0)	0.855
3–4	2415	(91.4)	842	(93.0)		840	(92.8)	842	(93.0)	
PSA										
< 10	1292	(48.9)	422	(46.6)	0.011	444	(49.1)	422	(46.6)	0.390
10–19.9	744	(28.1)	231	(25.5)		234	(25.9)	231	(25.5)	
≥ 20	607	(23.0)	252	(27.8)		227	(25.1)	252	(27.8)	
Gleason score										
6–7	1294	(49.0)	377	(41.7)	0.000146	401	(44.3)	377	(41.7)	0.254
8–10	1349	(51.0)	528	(58.3)		504	(55.7)	528	(58.3)	
Number of LN dissection										
1–9	1259	(47.6)	476	(52.6)	0.010	483	(53.4)	476	(52.6)	0.742
10 ≤	1384	(52.4)	429	(47.4)		422	(46.6)	429	(47.4)	
Number of positive LN										
1–3	2340	(88.5)	802	(88.6)	0.946	804	(88.8)	802	(88.6)	0.882
4 ≤	303	(11.5)	103	(11.4)		101	(11.2)	103	(11.4)	
Lymph node ratio (LNR)										
≤ 6%	361	(13.7)	116	(12.8)	0.522	117	(12.9)	116	(12.8)	0.944
7% ≤	2282	(86.3)	789	(87.2)		788	(87.1)	789	(87.2)	
Insurance										
Uninsured	2085	(78.9)	714	(78.9)	0.942	717	(79.2)	714	(78.9)	0.912
Insured	48	(1.8)	18	(2.0)		20	(2.2)	18	(2.0)	
Unknown	510	(19.3)	173	(19.1)		168	(18.6)	173	(19.1)	
Marriage										
Married	1877	(71.0)	645	(71.3)	0.162	626	(69.2)	645	(71.3)	0.609
Others	641	(24.3)	204	(22.5)		217	(24.0)	204	(22.5)	
Unknown	125	(4.7)	56	(6.2)		62	(6.9)	56	(6.2)	

Propensity-score matching was performed using optimal matching algorithm.

^*^Pearson Chi-square analysis

Abbreviations: ART, adjuvant radiotherapy; PSA, prostate-specific antigen; LN, lymph node; LNR, lymph node ratio.

### Prognostic impact of ART before propensity-score matching

**Figure 1:**
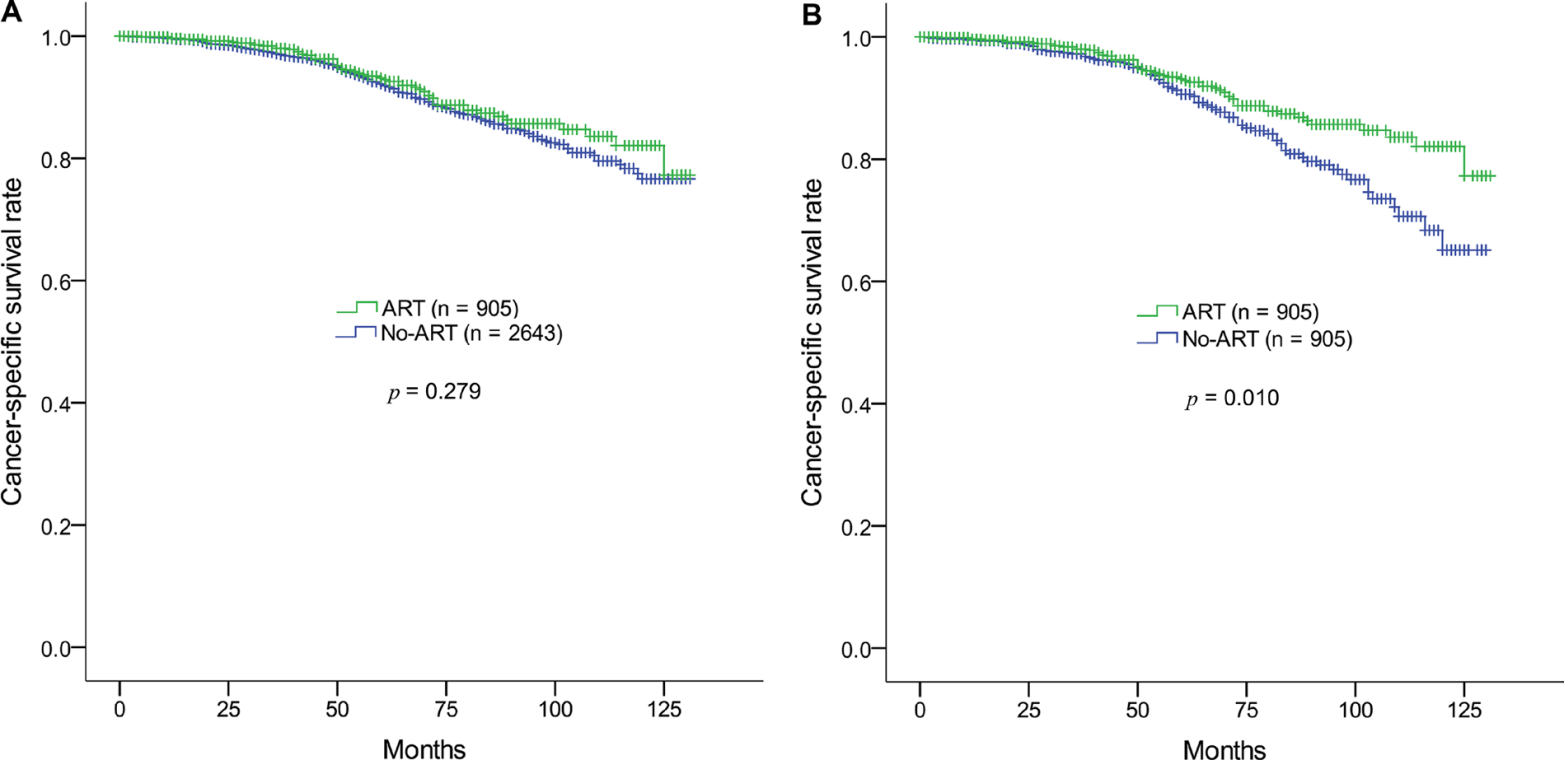
Kaplan-Meier survival estimates of cancer-specific survival rates in the ART and non-ART groups in prostate adenocarcinoma (pN1M0) after radical prostatectomy (**A**) Before and (**B**) after propensity-score matching. ART, adjuvant radiotherapy.

The Kaplan-Meier survival curve showed no CSS benefit as a result of ART (*p* = 0.279) (Figure 1A). The 5-year CSS rates in the no-ART and ART groups were 92.5% and 93.2%, and the 10-year CSS rates were 76.6% and 82.1%, respectively. Backward stepwise Cox multivariate regression incorporating all variables identified the following significant prognostic factors: ART (*p* = 0.036), number of positive LNs (*p* = 0.000052), LNR (*p* = 0.000003), Gleason score (*p* < 0.000001), and T categories (*p* = 0.005).

### Prognostic impact of ART after propensity-score matching

After propensity-score matching, the ART and no-ART groups each contained 905 patients. There were no statistic differences between any of the variables, except race (Table 1). Kaplan-Meier survival analysis compared by the log-rank test demonstrated that ART significantly improved the CSS rate (*p* = 0.010) (Figure 1B). Kaplan-Meier survival curves remained similar until 5 years after diagnosis; the 5-year CSS rates in the no-ART and ART groups were 91.5% and 93.2%, respectively. However, after 5 years, the difference between the survival curves increased, resulting in 10-year CSS rate of 65.1% and 82.1% in the no-ART and ART groups, respectively.

### The cut-off points of LNR for CSS rate before and after propensity-score matching

The estimated cut-off points of LNR for CSS rate were 33% (*p* < 0.000001) and 35% (*p* = 0.003) before and after propensity-score matching, respectively. Before propensity-score matching, the 10-year CSS rates in the dichotomized groups by the cut-off point (LNR < 33%, *n* = 2604 and LNR ≥ 33%, *n* = 944) were 81.2% and 70.7% (*p* < 0.000001), respectively. The 10-year CSS rates after the propensity-score matching using the cut-off point of 35% (LNR < 35%, *n* = 1413 and LNR ≥ 35%, *n* = 397) were 76.0% and 69.5% (*p* = 0.000134), respectively.

### Subgroup analyses

Subgroup analyses according to prognostic variables were performed. Throughout the subgroups analyses, ART consistently increased CSS rates (Table 2). Specifically, patients below 65 years of age (*p* = 0.010), with T3–4 category cancers (*p* = 0.009), a histology grade 3–4 (*p* = 0.017), fewer than 10 LN dissections (*p* = 0.013), or with an LNR of 7% or higher (*p* = 0.007) achieved significant CSS benefits as a result of ART.

**Table 2:** Comparison of 10-year cancer-specific survival rate in prostate adenocarcinoma after radical prostatectomy between the non-ART and ART groups after propensity-score matching

Characteristics	No-ART		ART		*P* value^*^
	10-year CSS (%)	95% CI	10-year CSS (%)	95% CI	
All	65.1	(55.8–76.0)	82.1	(76.7–87.8)	0.010
Age					
< 65	60.5	(48.7–75.0)	81.2	(74.6–88.3)	0.010
≥ 65	76.3	(66.0–88.2)	84.2	(75.0–94.4)	0.435
Race					
White	62.2	(51.7–74.7)	80.9	(74.7–87.6)	0.016
Black	79.2	(67.5–92.9)	94.8	(87.7–100.0)	0.035
Others	NA		69.9	(48.6–100.0)	0.142
Year of diagnosis					
2004–2009	64.8	(55.5–75.7)	81.9	(76.4–87.7)	0.009
2010–2014	NA		NA		0.635
T stage					
1–2	63.3	(34.9–100.0)	85.7	(96.6–100.0)	0.573
3–4	64.4	(54.7–75.7)	81.7	(76.4–87.4)	0.009
Grade					
1–2	NA		NA		0.111
3–4	65.5	(56.1–76.4)	81.8	(76.4–87.6)	0.017
PSA					
< 10	66.0	(54.3–80.2)	83.5	(75.7–92.1)	0.034
10–19.9	68.5	(51.0–92.2)	80.8	(70.7–92.4)	0.863
≥ 20	60.3	(44.8–81.2)	81.4	(72.3–91.7)	0.041
Gleason score					
6–7	65.6	(46.0–93.4)	90.8	(84.2–97.9)	0.015
8–10	62.0	(52.7–72.9)	75.8	(68.2–84.1)	0.067
Number of LN dissection					
1–9	64.5	(53.5–77.7)	85.8	(80.4–91.6)	0.013
10 ≤	68.8	(55.1–86.0)	76.5	(66.5–88.1)	0.283
Number of positive LN					
1–3	67.3	(57.2–79.2)	82.9	(77.2–89.0)	0.061
4 ≤	47.3	(31.5–70.9)	74.7	(61.2–91.4)	0.040
Lymph node ratio (LNR)					
≤ 6%	NA		63.2	(43.4-92.0)	0.941
7% ≤	65.2	(55.8–76.2)	84.0	(78.7–89.7)	0.007
Insurance					
Insured	NA		NA		0.381
Uninsured	NA		NA		0.425
Unknown	64.8	(54.9–76.4)	83.1	(76.7–90.0)	0.006
Marriage					
Married	62.7	(51.1–76.9)	81.6	(75.4–88.4)	0.032
Others	72.1	(61.3–85.0)	83.0	(74.9–92.1)	0.315
Unknown	NA		NA		0.333

^*^ Kaplan-Meier estimates were compared using log-rank test.

Abbreviations: ART, adjuvant radiotherapy; CSS, cancer-specific survival; CI, confidence interval; NA, not applicable; PSA, prostate-specific antigen; LN, lymph node; LNR, lymph node ratio.

Multivariate analysis using a backward stepwise regression was performed. The final Cox multiple regression model yielded the following significant prognostic factors: ART (hazard ratio [HR], 0.625; 95% confidence interval [CI], 0.444–0.879; *p* = 0.007), the number of positive LNs (*p* = 0.003), LNR (*p* = 0.018), T category (*p* = 0.028), and Gleason score (*p* < 0.000001) (Table 3).

**Table 3:** The final Cox multivariate analysis using a backward stepwise regression in prostate adenocarcinoma treated with radical prostatectomy

Characteristics	HR	(95% CI)	*P* value
Adjuvant radiotherapy (yes/no)	0.625	(0.444–0.879)	0.007
Number of positive lymph node (no.)	1.104	(1.034–1.178)	0.003
Lymph node ratio (LNR) (%)	1.009	(1.001–1.016)	0.018
T category (1–4)	1.445	(1.040–2.008)	0.028
Gleason score (2–10)	1.833	(1.516–2.217)	< 0.000001

Abbreviations: HR, hazard ratio; CI, confidence interval; LNR, lymph node ratio.

Subgroup analysis according to the number of positive LNs indicated that patients with up to 3 positive LNs did not show a statistically significant CSS benefit from ART (*p* = 0.061). In contrast, ART significantly increased the CSS rate for patients with 4 or more positive LNs (*p* = 0.040) (Table 2). Patients with 2 or more LN involvements or 3 or more positive LNs did not show any significant improvement in CSS with ART (*p* = 0.108 and *p* = 0.110, respectively).

**Figure 2:**
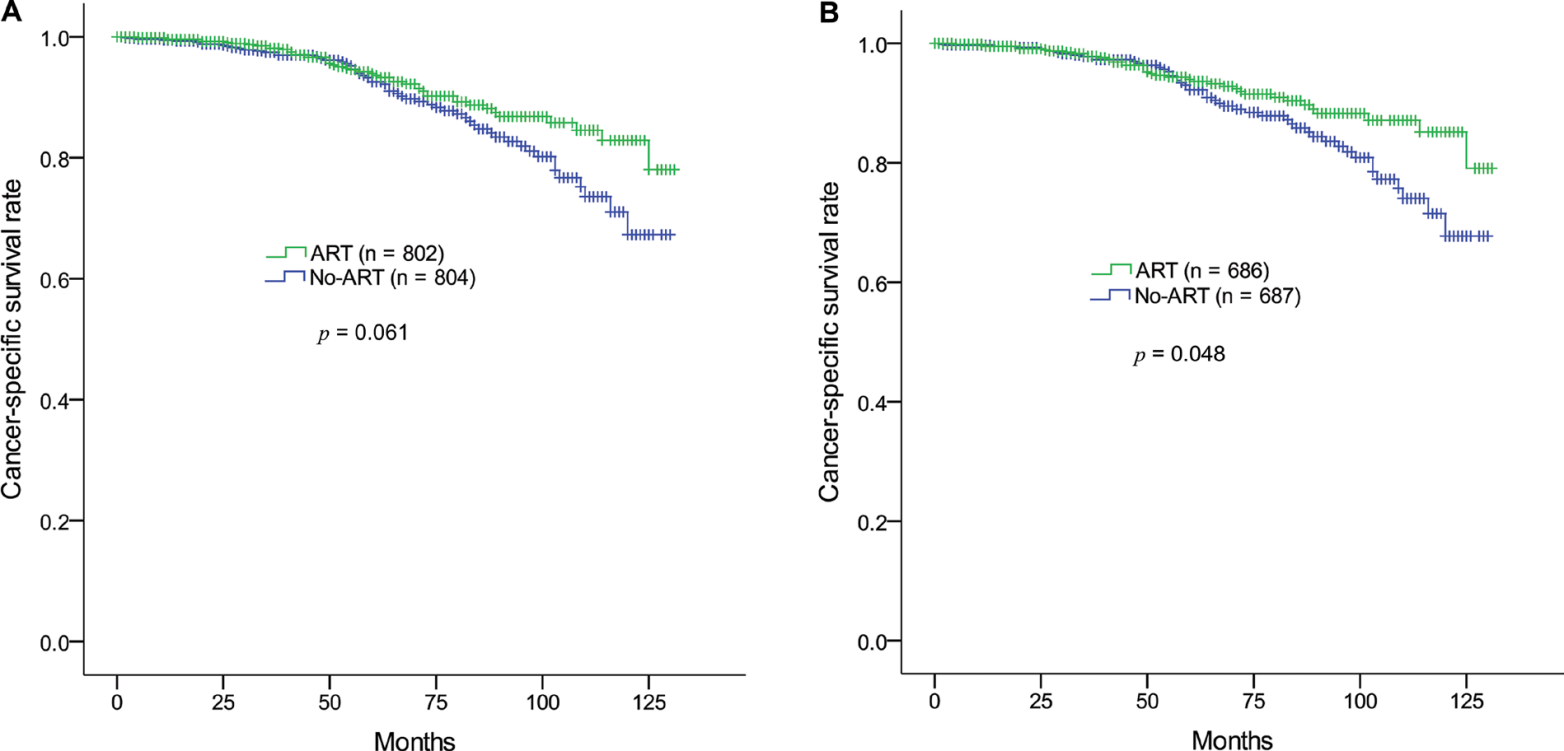
Kaplan-Meier survival estimates of cancer-specific survival rates in the ART and non-ART groups in prostate adenocarcinoma (pN1M0) after radical prostatectomy (**A**) The number of involved lymph nodes is 3 or less. (**B**) The number of involved lymph nodes is 3 or less and the lymph node ratio (100% × [number of positive LNs]/[number of dissected LNs]) is 7% or higher. ART, adjuvant radiotherapy; LN, lymph node.

Patients with up to 3 LN involvements were sequentially evaluated based on the number of positive LNs and the LNRs. The CSS benefit of ART manifested as the LNR increased; in patients with an LNR of 7% or higher, CSS rates increased significantly (Table 4; Figure 2). Even in patients with only 1 positive LN, treatment with ART yielded CSS benefits comparable to those who were not treated with ART (*p* = 0.034), provided the LNR was 7% or higher; the 10-year CSS rates in the no-ART and ART groups were 69.5% and 86.6%, respectively. However, patients with up to 3 LN involvements with an LNR of less than 7% showed no CSS difference regardless of the administration of ART. An LNR of less than 7% indicates at least 15 LN dissections in patients with only 1 positive LN, 29 LN dissections for 2 positive LNs, and 43 LN dissections for 3 positive LNs.

**Table 4:** Statistical significance of the cancer-specific survival benefit from adjuvant radiotherapy (ART) according to the number of involved lymph nodes and lymph node ratio (LNR) in prostate adenocarcinoma after radical prostatectomy

LNR (%)	Number of involved lymph nodes					
	1		≤ 2		≤ 3	
	*P* value^†^	mLND	*P* value^†^	mLND	*P* value^†^	mLND
≥ 1	0.043^*^	≤ 100.0	0.056	≤ 200.0	0.061	≤ 300.0
≥ 2	0.043^*^	≤ 50.0	0.056	≤ 100.0	0.061	≤ 150.0
≥ 3	0.048^*^	≤ 33.3	0.060	≤ 66.7	0.065	≤ 100.0
≥ 4	0.073	≤ 25.0	0.087	≤ 50.0	0.089	≤ 75.0
≥ 5	0.058	≤ 20.0	0.072	≤ 40.0	0.076	≤ 60.0
≥ 6	0.075	≤ 16.7	0.088	≤ 33.3	0.091	≤ 50.0
≥ 7	0.034^*^	≤ 14.3	0.043^*^	≤ 28.6	0.048^*^	≤ 42.9
≥ 8	0.034^*^	≤ 12.5	0.036^*^	≤ 25.0	0.042^*^	≤ 37.5
≥ 9	0.019^*^	≤ 11.1	0.024^*^	≤ 22.2	0.030^*^	≤ 33.3
≥ 10	0.018^*^	≤ 10.0	0.022^*^	≤ 20.0	0.028^*^	≤ 30.0
≥ 11	0.015^*^	≤ 9.1	0.017^*^	≤ 18.2	0.024^*^	≤ 27.3
≥ 12	0.030^*^	≤ 8.3	0.032^*^	≤ 16.7	0.039^*^	≤ 25.0
≥ 13	0.030^*^	≤ 7.7	0.032^*^	≤ 15.4	0.030^*^	≤ 23.1
≥ 14	0.014^*^	≤ 7.1	0.026^*^	≤ 14.3	0.022^*^	≤ 21.4
≥ 15	0.008^*^	≤ 6.7	0.019^*^	≤ 13.3	0.016^*^	≤ 20.0

^†^ Comparison of cancer-specific survival rates in the no-ART and ART groups using Kaplan-Meier method and log-rank test.

^*^ Statistically significant with *P* value less than 0.05.

LNR (%): 100% × [number of involved lymph nodes]/[number of dissected lymph nodes]

mLND: The maximum number of lymph node dissection to satisfy the corresponding LNR with the maximum number of lymph node metastases.

Abbreviations: ART, adjuvant radiotherapy; LNR, lymph node ratio; mLND, the maximum number of lymph node dissection.

## DISCUSSION

In this population-based propensity-score matching study, the CSS benefit resulting from ART for prostate cancer patients with LN involvement depended on the number of positive LNs and the LNR. In cases of 4 or more positive LNs, ART improved the 10-year CSS rate from 47.3% to 74.7% (*p* = 0.040). Patients with up to 3 positive LNs showed a 10-year CSS benefit as a result of ART when the LNR is 7% or higher (*p* = 0.048). Even in patients with only 1 positive LN, ART showed a significant 10-year CSS benefit (69.5% vs. 86.6%, *p* = 0.034), provided the LNR is 7% or higher. An LNR of less than 7% indicates that the number of LN dissections is 15, 29, and 43 or more for 1, 2, and 3 positive LNs, respectively. Therefore, even in patients with limited numbers of positive LNs, who did not receive extensive LN dissection, a CSS benefit from ART may be observed. The CSS benefit after ART was pronounced 10 years after diagnosis; therefore, ART should be recommended for patients with a long life expectancy.

According to a study of the pattern of failure in prostate cancer patients with positive LNs, the rate of local and/or nodal recurrence was 30.5% among those who experienced clinical recurrence [22]. This result suggests that loco-regional treatment remains essential for patients with positive LNs. Moreover, since ADT is an effective systemic therapy that has been a standard treatment for moderate to high-risk patients, local treatments seem to increase the chance of a cure [23].

Although ART provides a definite survival benefit to prostate cancer patients with positive LNs, when limited numbers of LNs are involved, only patients with additional risk factors appear to benefit from ART. Abdollah et al. [16] demonstrated that in patients with up to 2 positive LNs, ART was only beneficial to patients who were at pT3b/T4 stage, Gleason score 7 to 10, or showed a positive surgical margin. However, LNR was not evaluated in this study.

In our study, when patients with up to 3 positive LNs were confined to T3–4 categories, Gleason score 7 to 10, and the number of examined LNs was 10 or less, ART showed a significant CSS benefit (data not shown). However, when analyzing cases with an LNR of 7% or higher, ART showed a significant CSS benefit regardless of the T category or Gleason score. Therefore, it is likely that adequate loco-regional treatments (e.g., LN dissection and ART) for each patient with LN involvements are as important as the histological characteristics of the cancer to improve the treatment outcome.

A study using the National Cancer Database demonstrated that ART increases the overall survival rate in LN positive prostate cancer [10] and that this benefit was evident irrespective of the number of involved LNs. However, this study did not investigate the association between the LNR and the benefit of ART for each stratum of the number of positive LNs.

Our study used LNR cut-off points for the CSS rate before and after propensity-score matching of 33% and 35%, respectively, similar to cut-off points reported in the literature [13]. Although these cut-off points optimally reflect the survival outcomes, the concept of the cut-off point of LNR for the CSS benefit of ART may not be the same as that of the CSS outcome itself. For example, patients with involved LNs with an LNR of between 7% and 35% may show an increased CSS rate compared to those with an LNR of higher than 35%, and may also achieve a significant CSS benefit from ART.

Several studies demonstrated that extensive pelvic LN dissection increased survival for prostate cancer patients with positive LNs [24, 25]. An autopsy-based study suggested that approximately 20 pelvic LNs may serve as a guideline to ensure a sufficient standard pelvic LN dissection [26]. For patients with only 1 LN involvement, 20 LN dissections are sufficient to achieve an LNR of less than 7%. Our study showed that at least 15 LN dissections are sufficient to achieve this LNR in patients with 1 positive LN; patients with 2 positive LNs are able to achieve an LNR of marginally less than 7% with 29 or more LN dissections. In cases of 3 or more positive LNs, however, extensive LN dissection to obtain an LNR of less than 7% is impractical. In our study, among patients with 3 positive LNs, only 2 achieved an LNR of less than 7% (2/129, 1.6%), and no patient with 4 or more positive LNs achieved an LNR below 7%.

These results suggest that the correlation between the number of positive LNs and an adverse CSS outcome might not only stem from the burden of positive LNs itself, but also from the high LNR owing to inevitably insufficient LN dissection. In contrast, a high LNR implies the possibility of undissected (underestimated) positive LNs in patients with a limited number of LN involvements due to incomplete LN dissection. Extensive LN dissection is associated with adverse factors, such as long operating time, massive blood loss, prolonged length of hospital stays, and a high risk of postoperative complications [19]. Nowadays, robotic surgery in prostate cancer is widespread. Compared to open surgery, extensive LN dissection is not commonly performed during robotic surgery. For these reasons, extensive LN dissection is rare in the United States [10]. As a result, ART should be recommended for patients with positive LN dissection, but without extensive LN dissection, even when only 1 LN is involved. Concurrently, patients who are expected to have a limited number (*n* ≤ 2) of LN metastases before radical prostatectomy may benefit from extensive LN dissection, therefore omitting ART.

In our study, the CSS benefit was pronounced 10 years after diagnosis, which reveals the advantage of salvage treatment, including salvage RT, after biochemical failure. These salvage treatments appear to be effective within 10 years from the time of diagnosis. After 10 years, the difference of CSS rates between the no-ART and ART groups was clear, suggesting that in patients with a long life expectancy, ART should be recommended as one of the primary treatments at the time of diagnosis.

Despite efforts to reduce selection bias using propensity-score matching, unavoidable bias related to the retrospective characteristics and the limitation of the dataset may exist. Even though the SEER database collected PSA and Gleason scores from 2004 onwards, other information, including extracapsular extension, surgical margin, and postoperative PSA, was not provided. Information relating to ADT, salvage treatment, biochemical failure, and loco-regional/distant metastatic recurrences was also not available, although most patients with positive LNs in this study are considered to have received ADT.

Notwithstanding these limitations, this population-based propensity-score matching study demonstrated a definite CSS benefit to ART, even in patients with a limited number of positive LNs provided the LNR is 7% or higher. This finding may provide precise and effective loco-regional treatments for each prostate cancer patient with positive LNs.

In conclusion, prostate cancer patients with up to 3 positive LNs after RP achieved a 10-year CSS benefit from ART provided the LNR is 7% or higher (less than 15, 29, and 43 LN dissections in cases of 1, 2, and 3 positive LNs, respectively). Patients with 4 or more positive LNs obtained a CSS benefit from ART regardless of their LNR. Therefore, to determine the necessity of administrating ART after RP, the number of involved LNs and the LNR should be jointly considered.

## MATERIALS AND METHODS

### Patients

SEER 18 registries were used in this study. As the patient population of the data set is de-identified, Institutional Review Board approval was not applicable. From 2004 to 2014, men aged 19 years and older, who were diagnosed with pathologically confirmed primary prostate adenocarcinoma (International Classification of Diseases for Oncology ICD-O-3, C61.9/World Health Organization 2008, 8140/3), were included in this study. TNM categories were adjusted according to the American Joint Committee on Cancer's staging manual (8th edition), and patients in N1M0 categories were selected. All patients underwent RP (surgery code 50). The number of dissected and involved LNs was determined; at least 1 LN was dissected and at least 1 LN was involved. All patients were included in the following two RT groups: (1) postoperative external beam RT (ART group) and (2) no, unknown, or refused RT (no-ART group). No patient received chemotherapy as a primary treatment. Patients who were diagnosed by death certificate or autopsy only, and who had no information on survival time, race, T category, histology grade, PSA, and Gleason score, were excluded.

### Statistical analysis

The patients in the ART and no-ART groups were compared using Pearson Chi-square analysis before and after propensity-score matching. In an attempt to reduce selection bias inherent in analyses of the no-ART and ART groups, the no-ART group was matched to cases at a ratio of 1:1 using a propensity-score optimal matching algorithm based on age, race, year of diagnosis, T category, histology grade, PSA, Gleason score, the number of dissected LNs, the number of involved LNs, insurance, and marital status. Propensity-score matching was carried out by the R statistical software version 3.4.1 (www.r-project.org) using the “MatchIt” and “optmatch” R packages. The cut-off points of LNR for CSS before and after propensity-score matching were examined using the “maxstat” R package. Other statistical calculations were performed using SPSS software version 22 (SPSS, Chicago, IL).

## Abbreviations

ADT: androgen-depravation therapy
ART: adjuvant radiotherapy
CSS: cancer-specific survival
LN: lymph node
LNR: lymph node ratio
PSA: prostate-specific antigen
RP: radical prostatectomy
RT: radiotherapy
SEER: Surveillance
